# Long-Term Controlled Growth Factor Release Using Layer-by-Layer Assembly for the Development of *In Vivo* Tissue-Engineered Blood Vessels

**DOI:** 10.1021/acsami.2c05988

**Published:** 2022-06-13

**Authors:** Febriyani F. R. Damanik, Carolien T. Rothuizen, Reshma Lalai, Sandhia Khoenkhoen, Clemens van Blitterswijk, Joris I. Rotmans, Lorenzo Moroni

**Affiliations:** † Tissue Regeneration Department, MIRA Institute for Biomedical Technology and Technical Medicine, University of Twente, Drienerlolaan 5, Zuidhorst 145, 7522 NB Enschede, The Netherlands; ‡ Department of Internal Medicine, Leiden University Medical Center, Albinusdreef 2, 2333 ZA Leiden, The Netherlands; § Faculty of Science, Radboud University, Heyendaalseweg 135, 6525 AJ Nijmegen, The Netherlands; ∥ Complex Tissue Regeneration Department, MERLN Institute for Technology Inspired Regenerative Medicine, Maastricht University, P.O. Box 616, 6200 MD Maastricht, The Netherlands

**Keywords:** in situ tissue engineering, *in vivo* bioreactor, tissue-engineered blood vessels, vascular access, layer-by-layer, growth factor release, biomaterials

## Abstract

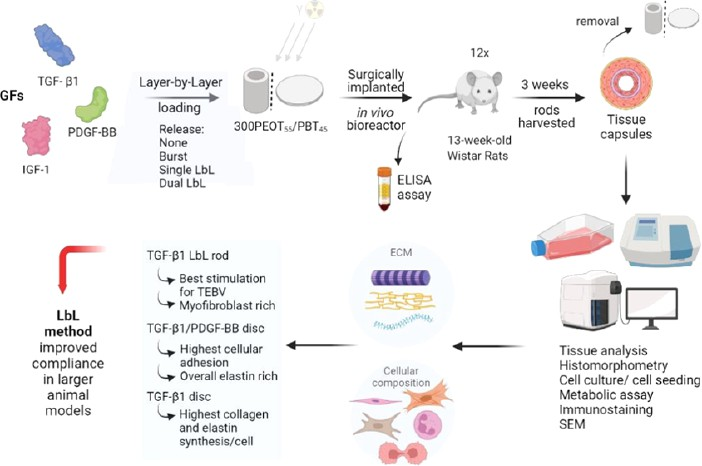

The development of a well-designed tissue-engineered blood vessel (TEBV) still remains a challenge. In recent years, approaches in which the host response to implanted biomaterials is used to generate vascular constructs within the patient’s body have gained increasing interest. The delivery of growth factors to these *in situ*-engineered vascular grafts might enhance myofibroblast recruitment and the secretion of essential extracellular matrix proteins, thereby optimizing their functional properties. Layer-by-layer (LbL) coating has emerged as an innovative technology for the controlled delivery of growth factors in tissue engineering applications. In this study, we combined the use of surface-etched polymeric rods with LbL coatings to control the delivery of TGF-β1, PDGF-BB, and IGF-1 and steer the foreign body response toward the formation of a functional vascular graft. Results showed that the regenerated tissue is composed of elastin, glycosaminoglycans, and circumferentially oriented collagen fibers, without calcification or systemic spill of the released growth factors. Functional controlled delivery was observed, whereas myofibroblast-rich tissue capsules were formed with enhanced collagen and elastin syntheses using TGF-β1 and TGF-β1/PDGF-BB releasing rods, when compared to control rods that were solely surface-engineered by chloroform etching. By combining our optimized LbL method and surface-engineered rods in an *in vivo* bioreactor approach, we could regulate the fate and ECM composition of *in situ*-engineered vascular grafts to create a successful *in vivo* vascular tissue-engineered replacement.

## Introduction

Competent and durable tissue-engineered blood vessels (TEBVs) have been the aim of many researchers in the broad field of vascular tissue engineering.^1,2^ TEBVs can serve as vascular substitutes for peripheral bypass or hemodialysis vascular access.^3,4^ The classical tissue engineering approach involves seeding vascular cells to synthetic scaffolds *in vitro*.^5^ Limitations of such an approach include bioreactor-based, expensive, laborious, and time-consuming procedures that are required to grow vital and sterile blood vessels.^6,7^ Hence, developing a suitable biological vascular graft with reliable function and durability, which eliminates the need for a bioreactor-based scaffold cellularization process, can be beneficial.

We previously proposed an *in vivo* TEBV that can serve as a vascular access graft for hemodialysis, whereby we use the body as a bioreactor. Previous *in vitro* studies preselected 6 from 56 cylindrical-shaped rods with engineered surfaces to be tested *in vivo* in rats,^8,9^ whereby a fibrocellular tissue capsule forms around it via foreign body response and creates the basis for the TEBV. Rothuizen et al. evaluated and selected a fibrocellular capsule formed by chloroform-treated rods as the best functional TEBV for its highly ordered collagen fiber distribution and mechanical properties. These *in vivo* results were supported by *in vitro* results, showing chloroform treatment to produce surface features that triggered functional properties of a blood vessel, and hence a suitable platform for the *in vivo* TEBV.

Nevertheless, the extracellular matrix (ECM) composition of such *in situ*-engineered tissues could still be improved by providing additional components in an effort to mimic a native blood vessel as closely as possible. For this purpose, the application of growth factors and/or cytokines offers an attractive option to improve the TEBV composition. Potential candidate growth factors include transforming growth factor β1 (TGF-β1), as TGF-β is a key enhancer of collagen and elastin secretion,^10,11^ thereby playing an important role in tissue capsule development.^12^ Furthermore, TGF-β triggers the differentiation of fibroblasts in tissue capsules to α-smooth muscle actin (α-SMA) expressing myofibroblasts.^13,14^ In addition, conjugation of TGF-β1 on hydrogels provided signaling and contractile functions of mesenchymal stromal cell-based vascular constructs.^15^ Platelet-derived growth factor (PDGF-BB) is another promising candidate to incorporate in cylindrical rods as PDGF-BB is a major mediator of foreign body response, involved in the early stage of myofibroblast differentiation^16^ and proliferation of tissue capsule cells.^17^ In addition, insulin growth factor 1 (IGF-1) could be used to stimulate elastin and collagen expressions^18,19^ in tissue capsules. IGF-1 has been shown to enhance the recruitment of bone marrow stromal cells to the injury sites, which stimulated muscle regeneration.^20^

To apply growth factors in tissue engineering approaches, different drug delivery systems used scaffolds as supports for their site of delivery.^21^ The various functionalities of such a delivery system can be to signal targeted cells to the site of implantation, vasculogenesis, or angiogenesis, following functional tissue regeneration.^22^ Recently, various studies have used a controlled drug delivery system to help regulate the inflammatory response, as well as the proliferation of targeted cells for tissue regeneration.^23−26^ Chemical additives, such as retinoic acid, can indirectly influence the immune response and enhance tissue regeneration.^27^ Chemokines and cytokines are used as a tissue engineering tool to orchestrate an optimum immune response to attain tissue repair,^25^ while growth factor delivery systems provide major biological signals for successful tissue regeneration.^28,29^

Many studies have been performed to embed growth factors into substrates for tissue regeneration.^30^ Due to the unstable nature and short lifetime of these growth factors, non-harsh efficient methods to embed them are necessary to provide successful tissue regeneration. Layer-by-layer (LbL) is a simple and flexible technique to incorporate biological molecules to facilitate tissue regeneration. Keeney et al. used the LbL technique to incorporate macrophage chemotactic protein 1 (MCP-1) on orthopedic implants to modulate their immune response upon implantation.^31^ Shah et al. used the LbL method to fabricate polyelectrolyte multilayer films releasing a controlled amount of incorporated multiple growth factors.^32^ Wound healing and foreign body response (FBR) are mediated by a complex cascade of endogenous growth factors, and preclinical research have documented the synergistic benefit of using multiple growth factors.^33,34^ Loading efficiency and controlled release are also major factors to be considered to provide cost-effective applications. Previously, we optimized an LbL method to embed different types of growth factors, namely, TGF-β1, PDGF-BB, and IGF-1, in a single or dual controlled release manner for up to 3 weeks.^35,36^ We examined the functionality of the embedded growth factors on their ability to affect proliferation, secretion of ECM proteins found in native blood vessels, namely, collagen and elastin, as well as morphological changes to exposed cells.

Hence, in this study, we implanted LbL-coated rods in adult Wistar rats, acting as cylindrical templates for the modulation of FBR into a fibrocellular tissue capsule with the capacity to transdifferentiate into a blood vessel-like structure upon implantation in the vasculature by exploiting an *in vivo* bioreactor strategy. We evaluated the *in vivo* TEBV produced histologically using immunohistochemistry and morphometry to determine the composition of the tissue capsule formed from the developed drug delivery scaffolds. *In vivo* results were correlated with cell proliferation, morphology, metabolic activity, and secretion of essential ECM proteins *in vitro*.

## Materials and Methods

### Polymeric Rod and Disc Fabrication

Copolymeric rods were composed of poly(ethylene oxide terephthalate)/poly(butylene terephthalate) (PEOT/PBT)—300PEOT45PBT45; following an *a*PEOT*b*PBT*c* nomenclature, *a* is the molecular weight in g/mol of the PEG blocks added in the copolymerization, while *b* and *c* are the weight ratios of the PEOT and PBT blocks, respectively. Polymeric rods were manufactured with a Bioplotter device (Envisiontec GmbH), an XYZ plotter device as previously described by Moroni et al.^37^ Briefly, PEOT/PBT pellets were loaded into a steel cartridge and heated at 180–200 °C. Using computer-aided manufacturing software (CAM, PrimCAM), rods were fabricated by extrusion at 4–5 bars to achieve cylindrical fibers of 1.75 mm in diameter. Two-dimensional substrates of PEOT/PBT were made for *in vitro* studies. Polymeric discs of 500 μm thickness were made by a hot-embossed compression molding technique as previously described.^9^ Briefly, granules of PEOT/PBT were distributed inside circular punched 1 cm-diameter stainless steel molds between two functionalized silicon wafers (FDTS, Sigma-Aldrich). The stack was placed in a temperature hydraulic press (Fortune Holland) at 180 °C and 10 bars. After 5 min, the system was cooled to 60 °C and the pressure was released. The mold and wafer were manually separated to obtain smooth PEOT/PBT discs.

### Presurface Treatment and Sterilization

Presurface treatments of the polymeric substrates were conducted to optimize the surface roughness, area, and charge for efficient loading of growth factors, as described previously.^38^ Briefly, fabricated rods were etched with chloroform for 10 s. Rods were, then, washed with MilliQ water and further sonicated twice for 15 min each. After drying, oxygen plasma treatment was performed with a reactive ion etch system (Etch RIE Tetske, Nanolab, University Twente) at 100 mTorr and 100 W for 5 min. All rods were sterilized by γ-radiation (Synergy Health) with a dose of 1.60–1.94 Mrad of 60 Co irradiation.

### Layer-by-Layer Loading of Single and Dual Growth Factors (GFs)

A precoating of polyelectrolytes was done in a sequence of polyethylenimine/poly(styrenesulfonate) (PEI/PSS)_2_ to provide ample charge strength. Then, a sequence of growth factor/heparin (GF/Hep)_4_PEI, for which GF (TGF-β1, PDGF-BB, or IGF-1) can be active, was deposited for single release; (TGF-β1/Hep/PDGF-ββ/Hep)_4_PEI and (IGF-1/Hep)_4_PEI-(TGF-β1/Hep)_4_PEI were used for dual release as previously described.^38^ Briefly, solutions of PEI and PSS at 2 mg/mL in MilliQ water and Hep at 1 mg/mL in NaCl 0.15 M were prepared and sterilized through a 0.02 μm filter (Sigma-Aldrich, Germany). Growth factors (TGF-β1, PDGF-BB, IGF-1, R&D Systems) were dissolved in 0.1% bovine serum albumin (BSA) in 1× phosphate buffer solution (PBS), at concentrations of 40, 40, and 100 ng/mL, respectively. A washing procedure with MilliQ water for a few seconds and gentle shaking was applied between different layer depositions. All processes were done in a sterile fume hood.

### Dip-Coating

The additive effect of controlled release was compared to a burst release obtained from dip-coated growth factor coating. Similar γ-radiated, chloroform-etched, oxygen plasma-treated 300PEOT55PBT45 rods were dipped for 1 min in a solution in 1× phosphate-buffered saline (PBS) of either activated TGF-β1 or PDGF-BB at a concentration of 40 ng/mL. Before further use, rods were dried for 5 min and meanwhile cooled on ice to prevent degradation of the GFs.

### Rat Model

All animal studies were approved by the Animal Care and Use Committee of the Leiden University Medical Center and were performed in accordance with the Dutch legislation. In total, twelve 13-week-old male Wistar rats were used. All surgical procedures were performed sterile under isoflurane anesthesia, and perfalgan (100 mg/kg) was injected for direct postoperative analgesia. In addition, perfalgan (2.7 mg/mL) was added to the drinking water up to one day after surgery. Per rat, four different types of rods were implanted subcutaneously on the ventral side. All rods were placed at ≥1.5 cm distance from each other. In short, a longitudinal subcutaneous pocket was bluntly created, and the rod was placed in the pocket. Subsequently, the opening was closed using 4–0 vicryl sutures (Johnson & Johnson, The Netherlands). All types of implanted rods are summarized in Table S1. All types of rods were implanted 6 times. Three weeks after implantation, the rods with tissue capsules deposited around it were collected and animals were sacrificed. At the time of implantation and sacrifice, blood was drawn and collected in heparin tubes and centrifuged and plasma was collected for enzyme-linked immunosorbent assay (ELISA) measurements. The collected plasma was quantified for TGF-β1, PDGF-BB, and IGF-1 with an ELISA assay following the kit instructions (DuoSet ELISA development kit, R&D Systems Europe Ltd.).

### Tissue Analysis

Tissues were fixed in 4% paraformaldehyde with the rods *in situ.* After removal of the rods, tissues were processed and embedded in paraffin. Serial sections of 4 μm were made. Sections were stained with hematoxylin–phloxine–saffron (HPS) for a general overview. The ECM was stained with picrosirius red for collagen, Alcian blue (pH 2.5) for glycosaminoglycans (GAGs), and Weigert’s elastin for elastin. Potential calcifications were evaluated with an Alizarin red staining. The cellular composition was evaluated with antibodies against α-smooth muscle actin (α-SMA, Dako, The Netherlands, 1:1000) for myofibroblasts, vimentin (Thermo Scientific, The Netherlands, 1:20 heat-induced citrate antigen retrieval) for fibroblasts, desmin (Thermo Scientific, The Netherlands, 1:50, heat-induced 0.1% trypsin antigen retrieval) for contractile smooth muscle cells, CD45 (Immunologic, The Netherlands, 1:150, heat-induced 0.1% trypsin antigen retrieval) for leukocytes, Von Willebrand factor (Dako, The Netherlands, 1:500, heat-induced 0.1% trypsin antigen retrieval) for endothelial cells, and Ki67 (BD Pharmingen, The Netherlands, 1:20, heat-induced citrate antigen retrieval) for proliferating cells, and visualized with 3,3′-diaminobenzadine (DAB). Fibroblasts, myofibroblasts, and contractile smooth muscle cells were differentiated as described elsewhere.^39^ All slides were visualized using a panoramic slide scanner (3-D Histec, Hungary) under a bright field. In addition, to differentiate fibrillar collagen, polarized light images were taken from the Picrosirius red-stained sections. After extrusion, the rods were stained with methylene blue to evaluate any possible adherence of tissues to the rods.

### Histomorphometry

The thickness of the fiber capsule TEBV positively stained for collagen, elastin, and glycosaminoglycans was measured using bright-field microscopy of all sections with CaseViewer (3DHISTECH Ltd.). Quantification was done with CaseViewer on at least eight measurements per section, for six sections. The thickness was measured using the “measure length” option, by defining a starting and an end point; the length was displayed on the connecting line. The total area was measured using the annotation mode. The “Free hand linear” tool was used to demarcate the total positive area. Thereafter, the area was displayed on the “Annotation panel”. Differentiation between tissue capsules and surrounding tissues for the measurements could clearly be made. Whereas the matrix of the tissue capsule was densely packed, the surrounding tissue was composed of a loosely packed matrix.

The quantification of the cells stained with α-SMA, vimentin, desmin, CD45, von Willebrand factor, and Ki67 was done as follows. The ECM of the tissue capsule was demarcated using the “Free hand linear” tool, creating an annotation with a total perimeter of ±2500 μm. This annotation was exported to 3D HISTECH’s Slide Converter, to convert the .mrxs file to a .tif file. The following specifications were used in the properties: automatic TIFF type, uncompressed 8-bit TIFF compression, Raw Image for image processing, RGB FL slide color channel, and 1:1 zoom level. The .tif file was opened in Fiji (ImageJ). The stained cells were manually measured using the cell counter plugin. To use the cell counter, the annotation was initialized first; then, one counter was chosen (one type of cells to count) and a color with high contrast was selected to mark all stained cells. The total number of counted cells is shown in the Results section.

### Cell Culture and Cell Seeding

Adult rat dermal fibroblasts (aRDF, #R2320, ScienCell Research Laboratories) were cultured with a basic culture medium comprising DMEM (Gibco), fetal bovine serum (10%, Lonza), l-glutamine (2 mM, Gibco), and penicillin (100 U/mL) and streptomycin (100 mg/mL, Gibco). aRDF were expanded at an initial seeding density of 5000 cells/cm^2^ in a culture medium and refreshed every 2–3 days. Cells were harvested at 80–90% confluency before trypsinization for cell seeding. Studies were performed with a cell seeding density of 2500 cells in a volume of 250 μL. Cultures were refreshed with the culture medium on days 1 and 4. All cell experiments were conducted in a 5% CO_2_ humid atmosphere at 37 °C.

### Metabolic Activity, Cell Proliferation, and GAG Assay

Metabolic activity was measured with the PrestoBlue reagent (Invitrogen). Samples (*n* = 6) were rinsed with 1× PBS (Gibco) and incubated with PrestoBlue with a culture medium (1:10) for 30 min. The solution was collected and analyzed at excitation and emission wavelengths of 570 and 600 nm, respectively. Total DNA was detected with the CyQuant cell proliferation assay kit (Molecular Probes) to assess cell attachment and proliferation on days 1, 4, and 7. Briefly, samples (*n* = 3) were rinsed gently with 1× PBS twice, placed into a 500 μL Eppendorf tube, and frozen at −80 °C. After three times freeze-thawing, 1× lysis buffer was added to the samples at room temperature (RT) for 1 h. For day 4 and day 7 samples, an additional overnight incubation was done at 56 °C in a Tris-EDTA-buffered solution (1 mg/mL proteinase K, 18.5 lg/mL pepstatin A, and 1 lg/mL iodoacetamide, Sigma-Aldrich). All samples were incubated for an additional 1 h with the lysis buffer RNase. Subsequently, cell lysate and CyQuant GR dye (1×) were mixed 1:1 in a black 96-well plate and placed in the dark for 15 min. Fluorescence was detected at excitation and emission wavelengths of 480 and 520 nm, respectively, with a spectrophotometer (The VICTOR3 Multilabel Plate Reader Perkin Elmer Corporation). The GAG amount was measured spectrophotometrically (EL 312e Bio-TEK Instruments) after reaction with dimethylmethylene blue dye (DMMB, Sigma-Aldrich) at 520 nm absorbance. The final quantification was calculated using a standard of chondroitin sulfate B (Sigma-Aldrich).

### Sircol and Fastin Assay for Collagen and Elastin Expression

Collagen was extracted from specimens (*n* = 3) on day 4 and day 7 using cold acid pepsin (0.1 mg/mL 0.5 M acetic acid) and left overnight at 4 °C. Collagen isolation and concentration were done before adding the SirCol dye reagent. The assay was conducted following the picosirius red-based colorimetric SirCol collagen dye binding assay kit (Biocolor Ltd.), and readings were collected at 540 nm. Rods (*n* = 3) for elastin quantification were first heated for 1 h at 100 °C with oxalic acid (0.25 M) to extract α-elastin. Further elastin quantification was obtained according to the Fastin elastin assay kit (Biocolor Ltd.), measured at 513 nm.

### Scanning Electron Microscopy and Immunostaining

Chemicals were bought from Sigma-Aldrich, unless differently specified. Samples (*n* = 4) were rinsed with PBS and fixed with 4% paraformaldehyde for 30 min at RT. After washing with PBS, samples (*n* = 2) were dehydrated with a sequential series of 70–80–90–100% ethanol solutions, 30 min per step. After dehydration, samples were dried with a critical point dry setup (CPD 030 Critical Point Dryer, Leica) and then gold-sputtered at 40 mA and 100 mTorr for 30 s. Cell morphology was investigated using a Philips XL30 ESEM-FEG SEM at 10 kV and a working distance of 10 mm.

For other polymeric discs (*n* = 2), attached cells were permeabilized and blocked with TBP buffer (0.1% Triton X-100, 0.5% bovine serum albumin in PBS) overnight at 4 °C. Cells were stained with monoclonal antiactin, α-smooth muscle (1:200) conjugated with goat antimouse Alexa Fluor 488 (Invitrogen, 1:1000) as the secondary antibody, Phalloidin-Texas red (Molecular Probes, 1:100), and 4′,6-diamidino-2-phenylindole (DAPI) (1:100) with three times washing steps in between. A Nanozoomer slide scanner equipped with a 40× objective (Hammamatsu) was used to acquire images.

### Statistical Analysis

All results were analyzed using GraphPad Prism 8.0.2. The results of the histomorphometry were obtained through manual digital image analysis and expressed as mean ± SEM. The results of the histomorphometry were tested using a one-way ANOVA with Tukey’s post hoc test. *P*-values of <0.05 were considered statistically significant. Statistical analysis of all *in vitro* results was conducted by GraphPad and expressed as mean ± standard deviation (s.d.). Biochemical assays were conducted with triplicate biological samples, unless differently stated. Statistical analysis was performed by two-way analysis of variance (ANOVA) with Bonferroni’s multiple-comparison test (*P* < 0.05), if not differently noted in the figure legends. For all figures, the following applies: *P < 0.05, **P < 0.01, ***P < 0.001.

## Results

### *In Vivo* Evaluation of Growth Factor Releasing Rods

**Figure 1:**
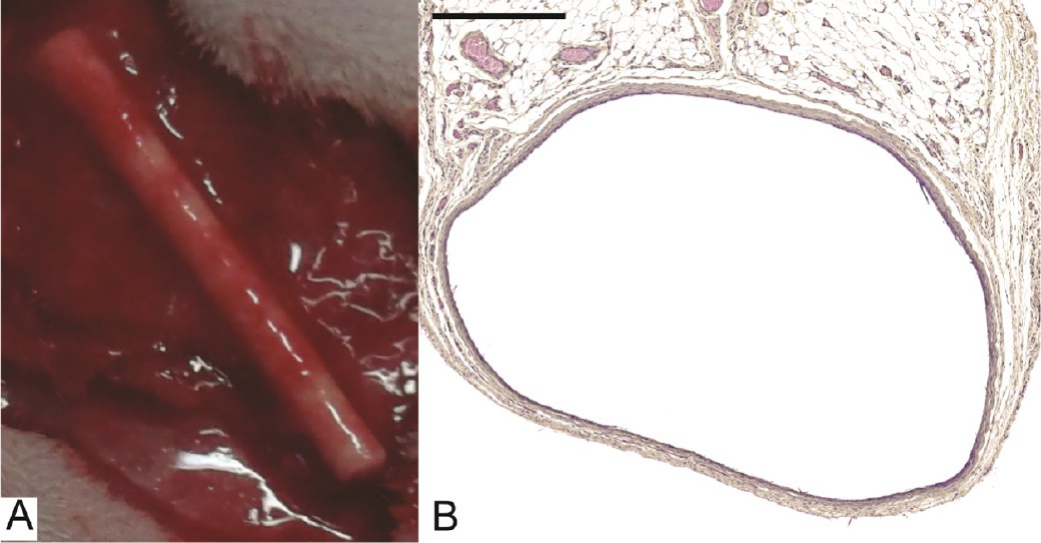
Autologous TEBV developed from the fibrocellular capsule. (A) Macroscopic picture of a rod encapsulated by a tissue capsule. The rod is still *in situ*. (B) Hematoxylin phloxin saffron-stained cross section of a tissue capsule formed around the TGF-β LbL rod, displaying the local thinner and thicker parts. Scale bar represents 500 μm.

**Figure 2:**
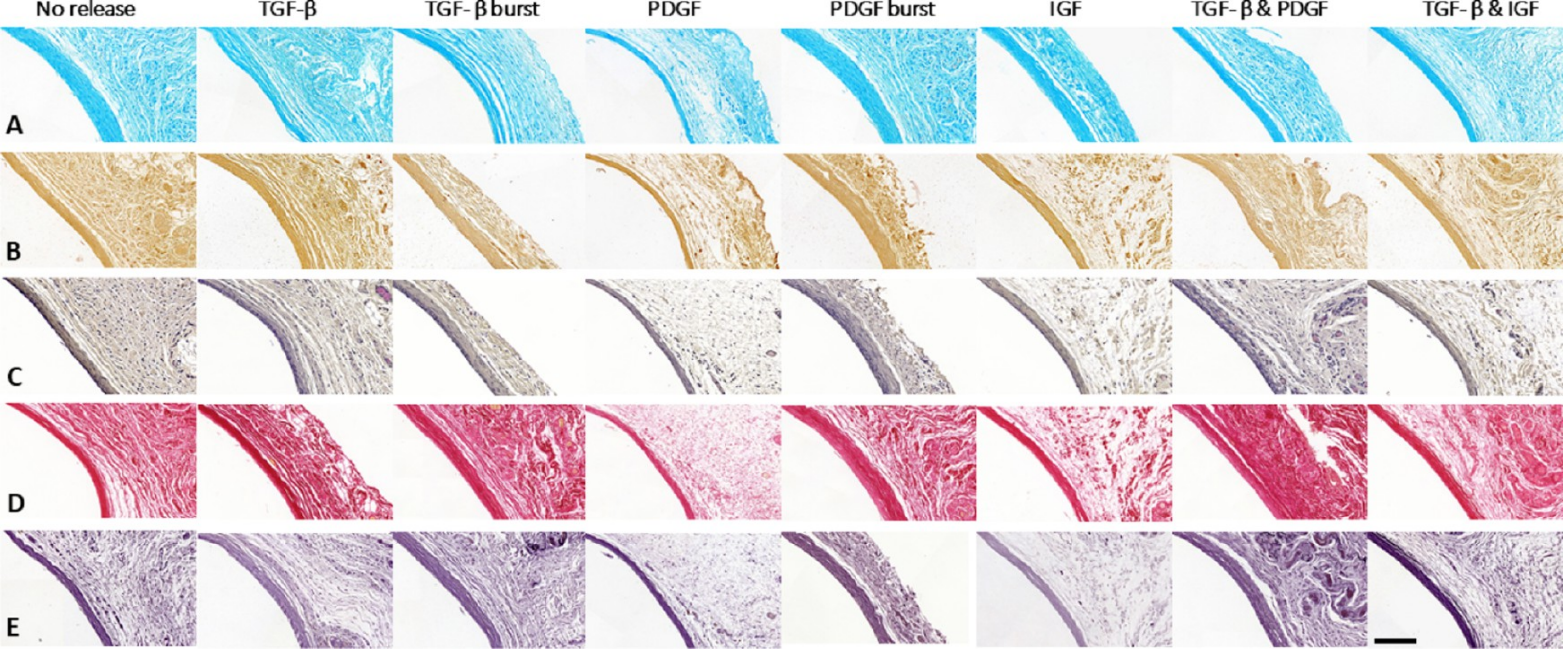
General overview of the ECM composition of the tissue capsule formed around different implanted rods. (A) Alcian blue staining for GAGs, (B) Alizarin red staining for calcification, (C) hematoxylin phloxin saffron for general overview, (D) Sirius red staining for fibrillar collagen, and (E) Weighert’s elastin stain for elastin. The ECM was largely composed of fibrillar collagen, elastin, and GAGs. There were no calcifications present. Scale bar represents 50 μm.

**Figure 3:**
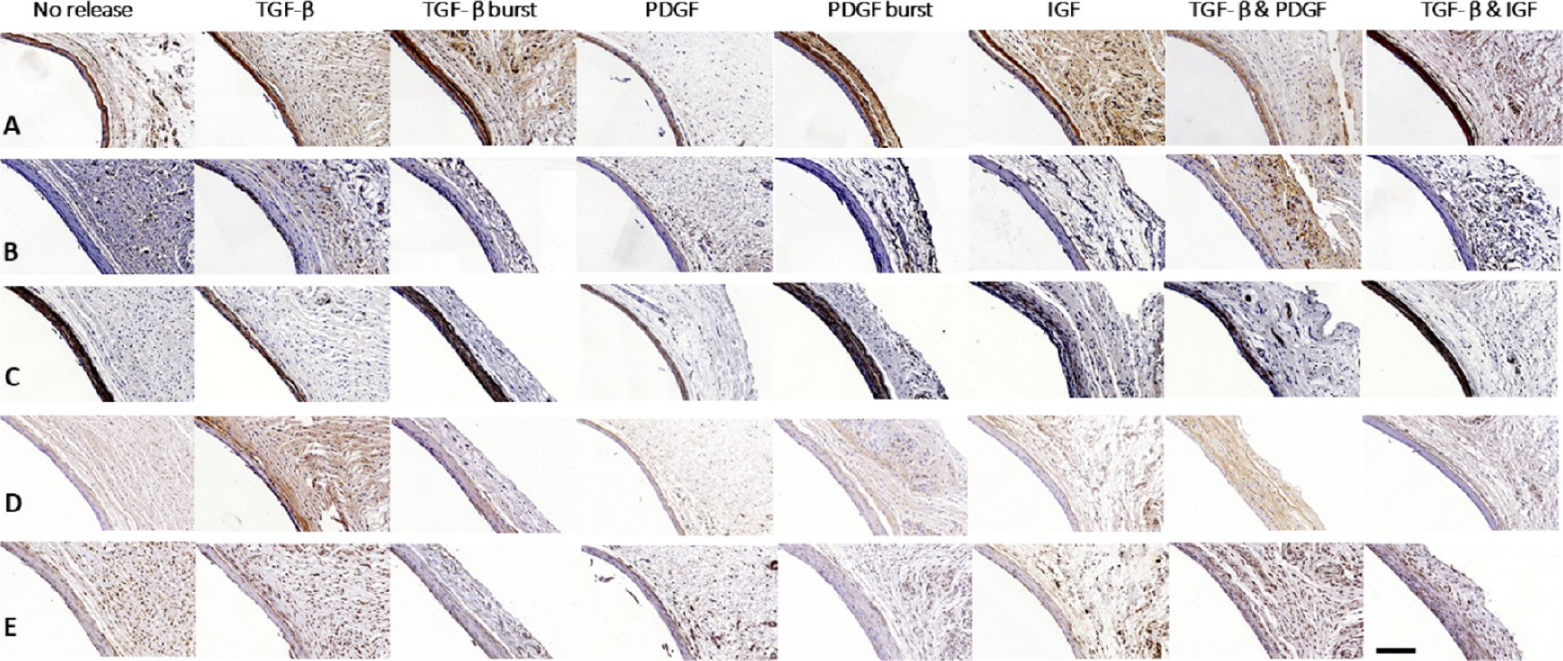
General overview of the cellular composition of the tissue capsule formed around implanted rods. (A) vWF staining for vascularization, (B) Ki67 for proliferating cells, (C) α-SMA for myofibroblast, (D) desmin for contractile smooth muscle cells, and (E) vimentin for fibroblast. The tissue capsules are mainly composed of fibroblasts and myofibroblasts. There are barely contractile smooth muscle cells present. All tissue capsules are well vascularized. Scale bar represents 50 μm.

**Figure 4:**
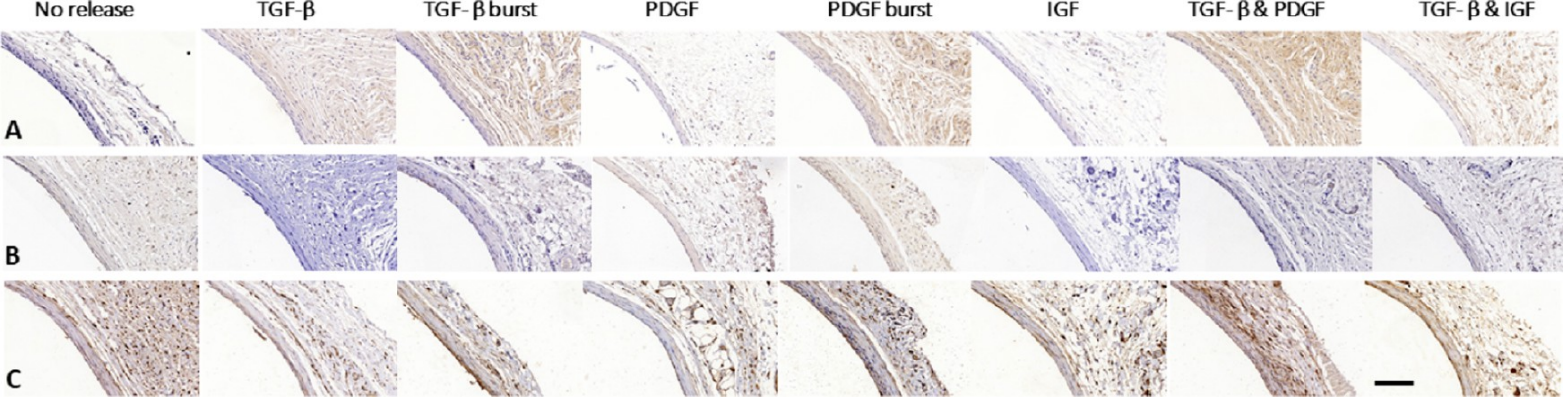
General overview of the immune cell composition of the tissue capsule formed around implanted rods. (A) CD45 staining for leukocytes. (B) CD68 for M1 macrophages, and (C) CD206 for M2 macrophages. Scale bar represents 50 μm.

Three weeks after implantation of the rods, all of them were encapsulated by a well-vascularized tissue capsule (Figure 1A). Rods with tissue capsules formed around them were successfully harvested. Macroscopically, no apparent differences were appreciated between the rods. Staining of the rods after removal of the fiber capsule revealed some tissue adhesion, which did not differ between different types of rods (data not shown). Tissue capsules formed around all types of rods were not completely homogeneous in wall thickness and displayed local thinner and thicker parts (Figure 1B). Generally, the matrix of the tissue capsules was made of circumferential GAGs (Figure 2A), orientated fibrillar collagen (Figure 2C,D), and elastin (Figure 2E). There were no calcifications (Figure 2B). All tissue capsules contained some proliferating cells (Figure 3B). Tissue capsules were prevalently composed of myofibroblasts (Figure 3C) and fibroblasts (Figure 3E). In contrast, little contractile smooth muscle cells were present (Figure 3D). Although most tissue capsules barely contained any leukocytes or foreign body giant cells (Figure 4A), in some tissue capsules leukocytes were present, particularly on the luminal side that was in contact with the polymeric rod. Furthermore, plasma collected before implantation and after explantation of the rods did not show significant concentration differences in growth factor levels, thus suggesting no systemic uptake of the growth factors after release (Figure S1).

Nonreleasing rods resulted in a few-cell-layer thick, mostly myofibroblast-rich tissue capsule. TGF-β LbL rods yielded remarkably thicker tissue capsules. Remarkably, tissue capsules formed around the LbL releasing rods were completely composed of myofibroblasts, whereas the TGF-β burst releasing rods only displayed myofibroblasts in the outer layer of the tissue capsule. In contrast, the inner layer of the tissue capsules formed around TGF-β burst releasing rods was composed of fibroblasts. Tissue capsules formed around both TGF-β LbL and burst releasing rods barely contained any leukocytes.

In contrast to the uniform morphology of the TGF-β LbL rods, the PDGF-BB LbL rods varied in morphology within the tissue capsules. Whereas some parts were completely composed of myofibroblasts, other parts only displayed myofibroblasts in the outer layer. In those regions, the inner layer was composed of both fibroblasts and some leukocytes. However, in the regions where myofibroblasts dominated, no leukocytes were observed. In contrast, their burst releasing controls resulted in a more uniform distribution in the tissue capsules, where the outer layer was composed of myofibroblasts, while the inner layer was composed of some leukocytes and many fibroblasts. This resembled the nonmyofibroblast-rich parts of the tissue capsules formed around the PDGF-BB LbL rods. Nevertheless, PDGF-BB LBL rods showed a higher amount of myofibroblasts than PDGF-BB burst release rods. Rods with the combined controlled release of PDGF-BB and TGF-β were also evaluated. This yielded rather uniformly distributed tissue capsules equally composed of myofibroblasts and fibroblasts. Very few leukocytes were observed. Next to TGF-β and PDGF-BB, IGF-1 controlled releasing rods were implanted. These rods were encapsulated by tissue capsules with an outer layer of myofibroblasts, an inner layer of fibroblasts, and barely any leukocytes. Surprisingly, the combination of controlled IGF-1 and TGF-β release did not seem to differ from controlled IGF-1 release alone, and thus contained less myofibroblasts than the single TGF-β LbL releasing rods.

When the TEBV thickness was analyzed, no significant difference in the GAG amount was noted among the different GF conditions compared to the control group (Figure S2A; no release = control group). GAGs were significantly increased in the PDGF-BB burst release condition compared to both the single LbL release of PDGF-BB and the dual release of PDGF-BB/TGF-β1. In the overall expression of GAGs across the TEBV area (Figure S2B), IGF-1 was a better candidate to enhance the expression of GAGs compared to the single LbL release of TGF-β1 and the dual LbL release of TGF-β1/IGF-1. A single release of IGF-1 significantly increased the expression of GAGs when compared to the TGF-β1 LbL release and to the dual TGF-β1/IGF-1 LbL release.

**Figure 5:**
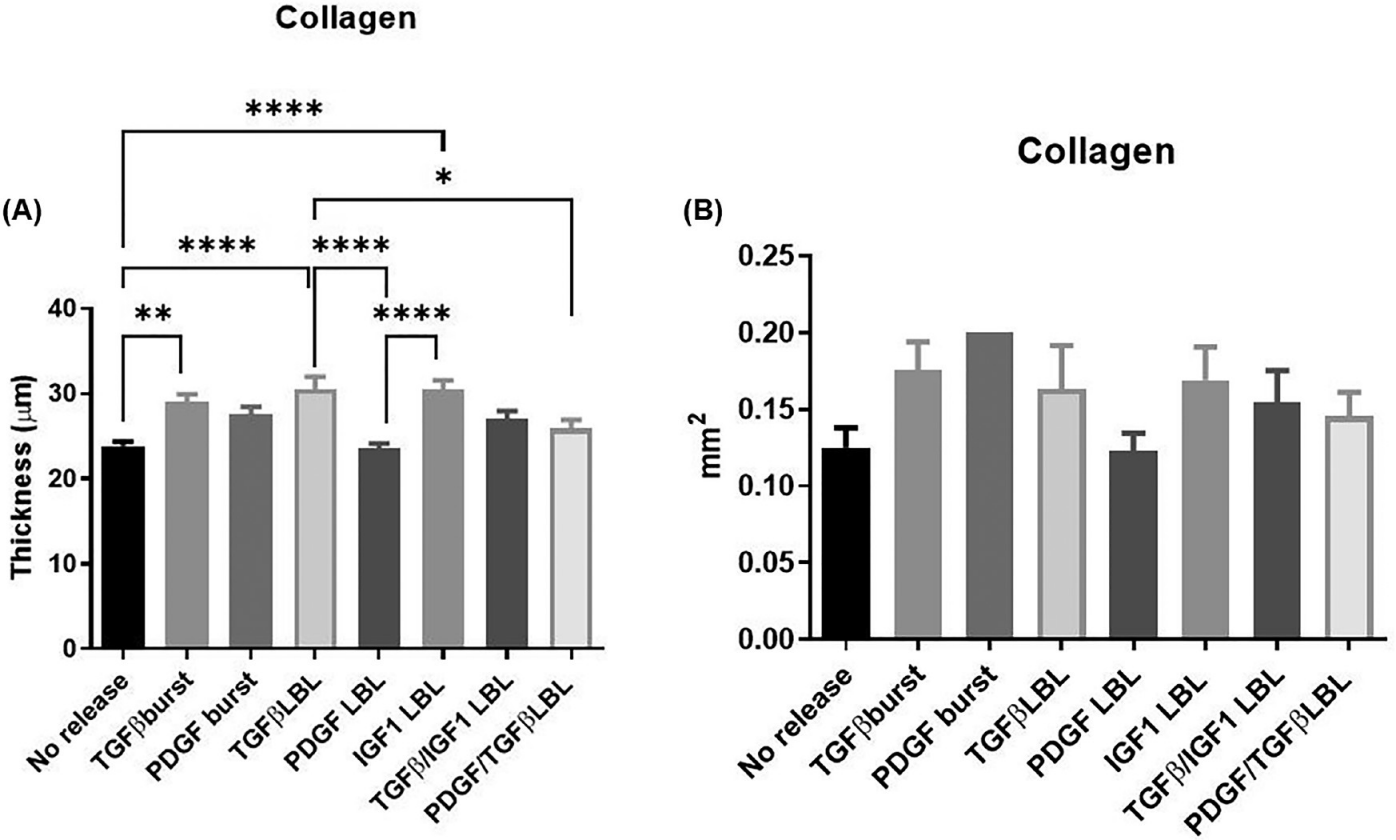
(A) Thickness (in μm) of positively stained ECM with picrosirius red for collagen. (B) The total area (in mm^2^) positively stained with picrosirius red for collagen is depicted. The expression of collagen was observed in the following conditions: control (no release), burst release (TGF-β1 and PDGF-BB), single layer-by-layer release (TGF-β1, PDGF-BB, and IGF-1), and dual layer-by-layer release (TGF-β1/IGF-1 and PDGF-BB/TGF-β1). Data were analyzed using ordinary one-way ANOVA followed by a post hoc analysis using Tukey’s test, and * indicates significance of *P* ≤ 0.05, **P ≤ 0.01, ***P ≤ 0.001, and ****P ≤ 0.0001.

When compared to the control group, a significant increase in collagen thickness was observed in conditions with single release of TGF-β1, independent of whether the release was burst or controlled by LbL; TGF-β1 LbL also induced a significantly higher collagen thickness compared to PDGF-BB LbL (Figure 5A). Besides, single LbL release of IGF-1 showed a significantly enhanced expression of collagen compared to the control and PDGF-BB LbL. TGF-β1 and IGF-1 have both been previously described as key enhancers in collagen secretion.^19,40^ Thereafter, all single LbL release conditions were compared, where increased collagen thickness was again associated with TGF-β1 and IGF-1 single LbL release. PDGF-BB LbL release was comparable to the control group. Conditions containing TGF-β1 were compared, where TGF-β1 LbL release was a better candidate for collagen expression than the dual LbL release containing TGF-β1 (Figure 5A). In the overall expression of collagen across the TEBV area, no significant differences were observed between the conditions (Figure 5B).

**Figure 6:**
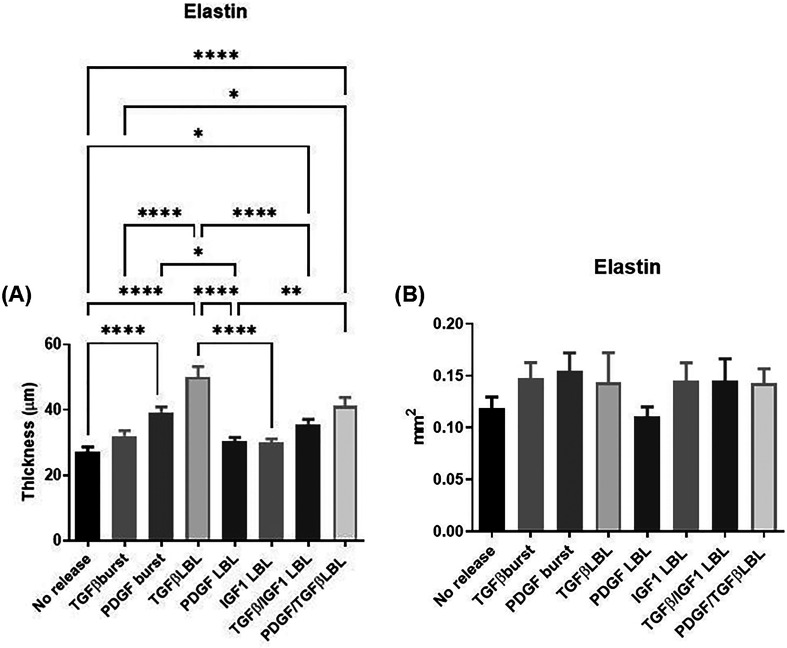
(A) Thickness (in μm) of positively stained ECM with Weigert’s stain for elastin. (B) The total area (in mm^2^) positively stained with Weigert’s stain for elastin is depicted. The expression of elastin was observed in the following conditions: control (no release), burst release (TGF-β1 and PDGF-BB), single layer-by-layer release (TGF-β1, PDGF-BB, and IGF-1), and dual layer-by-layer release (TGF-β1/IGF-1 and PDGF-BB/TGF-β1). Data was analyzed using ordinary one-way ANOVA followed by a post hoc analysis using Tukey’s test, and * indicates significance of *P* ≤ 0.05, **P ≤ 0.01, ***P ≤ 0.001, and ****P ≤ 0.0001.

Compared to the control, the release of TGF-β1 (single LbL as well as dual LbL release) showed a significantly increased expression of elastin thickness (Figure 6A). When all conditions containing TGF-β1 were compared, the single LbL release of TGF-β1 was a better candidate to promote elastin expression compared to the burst and dual LbL releases. PDGF-BB (burst and dual LbL releases) significantly enhanced the expression of elastin compared to the control. The single LbL release resulted in minimum stimulation comparable to the control. IGF-1 had previously been described as a key regulator in elastin expression.^18^ The comparison between the single LbL conditions showed that TGF-β1 remained the key enhancer for elastin expression.^41^ In the overall expression of elastin, no significant differences were found between the conditions (Figure 6B).

**Figure 7:**
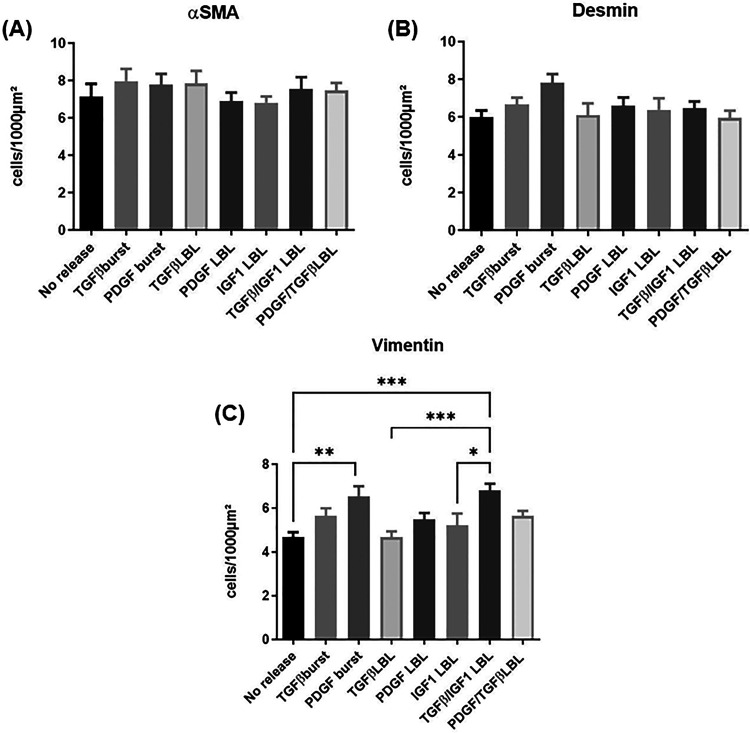
(A) The number of cells (per 1000 μm^2^) positively stained for α-smooth muscle actin is depicted, a differentiation marker for myofibroblasts. (B) The number of cells (per 1000 μm^2^) stained positively with the desmin antibody is depicted, which stains the intermediate filament protein expressed in contractile smooth muscle cells. (C) The number of cells (per 1000 μm^2^) positively stained for vimentin is depicted, which is an intermediate filament expressed in fibroblasts. The expressions of myofibroblasts, contractile smooth muscle cells, and fibroblasts were observed in the following conditions: control (no release), burst release (TGF-β1 and PDGF-BB), single layer-by-layer release (TGF-β1, PDGF-BB, and IGF-1), and dual layer-by-layer release (TGF-β1/IGF-1 and PDGF-BB/TGF-β1). Data was analyzed using ordinary one-way ANOVA followed by a post hoc analysis using Tukey’s test, and * indicates significance of *P* ≤ 0.05, **P ≤ 0.01, ***P ≤ 0.001, and ****P ≤ 0.0001.

No significant differences were found in the myofibroblast (Figure 7A) or contractile smooth muscle cell (Figure 7B) expression between the different conditions. In the vimentin antibody staining (Figure 7C), both PDGF-BB burst and dual TGF-β1/IGF-1 LbL release significantly enhanced the expression of myofibroblasts. Dual LbL release of TGF-β1/IGF-1 appeared to be a better condition than single LbL release of either TGF-β1 or IGF-1.

When compared to the control, a significant increase in leukocyte expression (CD45^+^ cells) was observed in the PDGF-BB burst release condition. No other significant differences were observed when compared to other conditions (Figure S3A). Similarly, no significant differences were found in the macrophage expression (CD68^+^ and CD206^+^ cells) in the different rod conditions (Figure S4). The overall expression of endothelial cells was stable in all conditions, as shown by von Willebrand factor antibody staining (Figure S3B). Furthermore, there were no differences found in the expression of proliferating cells compared to the control. However, there was a visible nonsignificantly increased expression in the PDGF-BB burst condition. When PDGF-BB-containing conditions were compared, a significantly increased expression was observed for PDGF-BB burst release (Figure S3C).

### *In Vitro* Analysis on Fibroblast Attachment and Metabolic Activity

*In vitro* studies were done to support and establish a relation to *in vivo* results of LbL rods and nonreleasing rods (control). Despite the lower cell attachment on all LbL rods compared to the control, there was no statistically significant difference seen on day 1 in cell attachment. On day 4, DNA quantification in control discs showed statistically higher fibroblast attachment compared to all LbL discs (Figure S5A). TGF-β1/IGF-1 discs had a statistically significantly higher cell attachment than all of the other LbL discs, with the exception of discs releasing PDGF-BB, namely, PDGF-BB alone and TGF-β1/PDGF-BB discs. On day 7, discs with PDGF-BB showed a higher cell attachment than the other LbL discs. The metabolic activity of cells attached to LbL discs was much higher than that of the control discs (Figure S5B). Cells attached to TGF-β1 discs showed the highest metabolic activity at all time points. Similar to TGF-β1 discs, cells found in TGF-β1/PDGF-BB provided a higher metabolic activity compared to all of the other discs, although statistically significantly lower than the cells in TGF-β1 discs on days 4 and 7. Cells found in all LbL discs supported a significantly higher metabolic activity compared to PDGF-BB discs at all time points, with the exception of day 4 in relation to TGF-β1/IGF-1. Similarly, with the exception of day 4, adhered cells in all LbL discs except for PDGF-BB provided a higher statistically significant metabolic activity than the control.

### *In Vitro* Cell Morphology and Myofibroblast Differentiation

**Figure 8:**
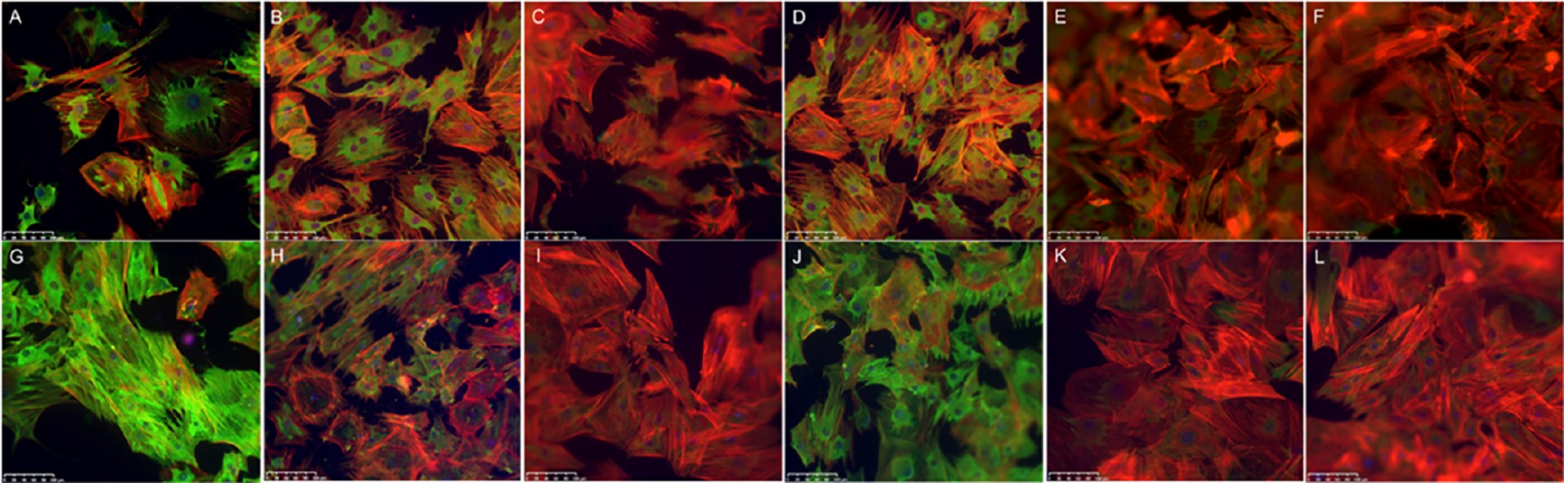
Immunostaining images on (A–F) day 4 and (G–L) day 7, showing α-smooth muscle actin (green), phalloidin (red), and DAPI (blue) on different discs. (A, G) Cells found in TGF-β1 LbL discs showed the majority of cells to be positive for α-smooth muscle actin and pronounced actin stress fibers. (B, H) PDGF-BB discs show cells with high α-smooth muscle actin positive cells and defined stressed actin fibers. (D, J) Cells attached to TGF-β1/PDGF-BB showed highly stressed actin fibers and increased α-smooth muscle actin expression on day 7. Cells attached to (C, I) IGF-1 and (E, K) TGF-β1/IGF-1 showed quite similar expressions to the (F, L) control. Scale bar is 100 μm.

Differences in cell morphology attached to different LbL discs and untreated discs (control) on day 1 were observed *in vitro* (Figure S6). TGF-β1 containing discs displayed square-like cells, while cells in PDGF-BB and IGF-1 discs were more elongated, similar to the control. Dual-release discs displayed a combination of squared and elongated morphology of the cells. Nevertheless, in all samples, cells seemed to maintain contact with one another. Immunostaining displayed fibroblasts that differentiated them from myofibroblasts characterized by stressed fiber actins (Figure 8). Cells on TGF-β1 discs were found to be more spread out with defined actin fibers on day 4 and highly expressed α-smooth muscle actin (α-SMA) staining at both time points. Expression of α-SMA was lower in cells found in IGF-1 containing discs, similar to cells in control discs, with some pronounced actin stress fibers on day 7. Cells on PDGF-BB discs displayed stressed actin fibers and pronounced secretion of α-SMA. For both time points, cells attached to TGF-β1/PDGF-BB discs showed abundant actin stress fibers and a high expression of α-SMA, with increasing differentiation of the myofibroblast population on day 7.

### LbL Discs’ Capability to Trigger Collagen, Elastin, and GAG Expression *In Vitro*

**Figure 9:**
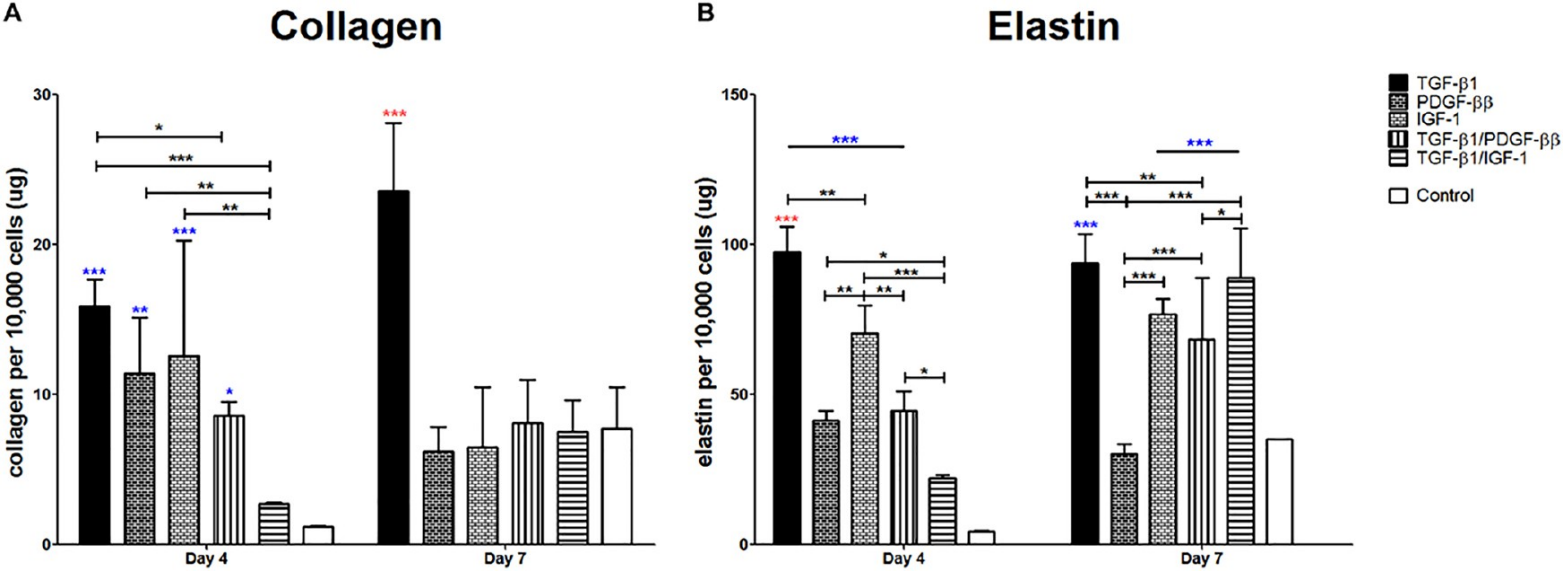
Protein secretion normalized by cell amount. Different discs are displayed by different bar patterns. Data are shown as mean ± s.d. (*n* = 3). (A) The collagen assay showed significantly higher collagen secretion per cell by LbL discs compared to the control on day 4, with the exception of TGF-β1/IGF-1 and TGF-β1 with the highest secretion on day 7. (B) Highest elastin secretion per cell was seen in TGF-β1 discs on day 4, and LbL discs were significantly higher than the control, with the exception of TGF-β1/IGF-1 discs on day 4 and PDGF-BB discs on day 7. Blue stars (*P < 0.05, **P < 0.01, ***P < 0.001) indicate statistical significance in comparison to the control, red stars designate the most optimal parameter from all treatment types, with the red # showing the second best, while the black stars indicate statistical differences between the different discs.

ECM proteins were isolated to analyze LbL discs’ capability of inducing collagen, elastin, and GAG secretion (Figures 9 and S7). Despite no significant difference in the total collagen secretion of LbL discs compared to the control on day 4, all LbL discs except for TGF-β1/IGF-1 displayed statistically higher collagen per cell expression than untreated discs. Cells found in TGF-β1 discs secreted statistically significantly higher amounts of collagen per cell than TGF-β1/PDGF-BB. On day 7, cells attached to TGF-β1 discs secreted statistically the highest amount of collagen, while other LbL discs secreted similar amounts compared to untreated discs. The total collagen secretion showed no difference in LbL discs compared to the control, with the exception of IGF-1 containing LbL discs, which secreted much less.

The secretion of elastin per cell was significantly higher in LbL discs than the control on day 4, with the exception of TGF-β1/IGF-1 containing discs. In contrast, cells found in IGF-1 releasing discs alone showed significantly higher elastin per cell production than the other discs, with the exception of TGF-β1 discs, showing the highest elastin secretion on day 7. On day 7, cells attached to discs containing either TGF-β1 or IGF-1 supported an increased elastin secretion. All LbL discs supported a statistically significantly higher secretion per cell than the control, with the exception of PDGF-BB discs. While the total elastin secretion was significantly the highest in TGF-β1/PDGF-BB, other LbL discs exhibited no statistical differences from the control (Figure S7).

GAG secretion normalized by the number of cells (Figure S8) was the highest in TGF-β1 discs at all time points. With the exception of TGF-β1 discs, cells found in TGF-β1/PDGF-BB discs provided the highest secretion than all of the other discs on day 1. However, at a later stage, IGF-1 containing discs supported a higher secretion than PDGF-BB-containing discs, but statistically significantly lower than TGF-β1 discs on days 4 and 7. GAG secretion in LbL discs was always statistically significantly higher than the control on days 1 and 4, while it was only significantly higher in TGF-β1 and IGF-1 containing LbL discs on day 7 than the untreated discs.

## Discussion

We investigated single- and dual-release LbL implants for their ability to affect tissue capsule composition for vascular tissue engineering using a rat model and studied their effect on cellular activity *in vitro*. We observed that controlled growth factor release can influence fibroblast attachment, morphology, differentiation, and matrix synthesis *in vitro*. When controlled releasing LbL rods were implanted *in vivo*, the cellular composition of the tissue capsule formed around it was modulated by the growth factors involved. TGF-β1 LbL implants generated the most favorable effect *in vitro* and *in vivo*, resulting in homogeneous collagen and myofibroblast-rich tissue capsules. Dual release of TGF-β1 combined with IGF-1 or PDGF-BB showed inferior results compared to single release of TGF-β1. In addition, tissues formed around all LbL controlled release differed from burst release controls, underlining the importance of controlled release.

Blood vessels consist of cells embedded in an ECM composed largely of proteins such as collagen, elastin, and GAGs, forming a concentric layered structure. Our *in vivo* studies showed that the matrix of tissue capsules has circumferentially orientated fibrillar collagen, elastin, and GAGs, with no calcification. These results are aligned with our previous studies^8,42^ and native blood vessel composition and alignment, where the collagen matrix is aligned in a spiral pattern along the axis of the vessel.^43^

### TGF-β1 Releasing Implants

TGF-β1 stimulates ECM deposition by increasing the collagen, fibronectin,^11,44^ and tropoelastin syntheses,^45,46^ all key matrix proteins in native blood vessels, and can attenuate matrix metalloproteinase (MMP)-mediated ECM degradation.^47^ Additionally, TGF-β1 drives differentiation of fibroblasts to myofibroblasts *in vivo*(48) and *in vitro*.^13^ Therefore, long-term release of TGF-β may enhance tissue-engineered blood vessel formation. Indeed, *in vivo*, tissue capsules formed around TGF-β1 LbL rods showed the highest density of myofibroblasts compared to all other growth factor releasing rods and control rods. *In vitro* studies further supported this by α-SMA staining, where TGF-β1 LbL discs were seen to have the highest population of myofibroblast differentiation. In contrast, a lower quantity of myofibroblasts was found in TGF-β1 burst releasing rods and only at the outer layer of the tissue capsule, underlining the capability of sustainable and efficient release on LbL rods, compared to burst release. In corroboration with this, in a previous study,^8^ dip-coated TGF-β1 rods showed no significant difference compared to uncoated rods.

Additionally, our *in vitro* results showed that cells attached to TGF-β1 LbL discs secreted the highest amount of collagen, elastin, and GAGs and displayed the highest rate of metabolic activity compared to all of the other discs, although these differences were not observed *in vivo*. This may be attributed to the reduced amount of cell attachment *in vitro*, as the increase in collagen compared to the other discs was only significant when corrected for cell number. This reduced cell attachment did not negatively affect tissue capsule formation *in vivo*. Rather, tissue capsules formed around TGF-β1 LbL rods resulted in the most favorable and homogenous tissue composition, rich in collagen and myofibroblasts. Myofibroblasts have been known to be plastic cells with a contractile apparatus and can synthesize and remodel ECM if stimulated.^49^ Additional high flow and circumferential stretch can trigger myofibroblasts to smooth muscle cell differentiation,^50^ which secrete collagen and elastin to consequently normalize the tension and hence trigger matrix synthesis.^51,52^ Moreover, tissue capsules rich in myofibroblasts have been shown to remodel to smooth muscle cell-rich constructs once placed in the vasculature.^53^ Hence, TGF-β1 LbL rods with a complete myofibroblast lining might provide the best stimulation for a potential tissue capsule to develop into a functional TEBV.

### PDGF-BB Releasing Implants

PDGF-BB stimulates chemotaxis and mitogenicity of, among others, leukocytes, fibroblasts, and smooth muscle cells and enhances collagen and proteoglycan synthesis.^54,55^ PDGF-BB has also been found to be a modulator of myofibroblast differentiation.^56,57^ In the present study, PDGF-BB LbL rods yielded an inhomogeneous tissue capsule, with parts with many myofibroblasts and other parts where the luminal area was populated with leukocytes and fibroblasts, and only the outer region was populated with myofibroblasts. Despite this nonhomogeneous population of the tissue composition, possibly due to the short half-life time of PDGF-BB of seconds, PDGF-BB LbL rods showed a higher quantity of myofibroblasts than PDGF-BB burst release rods. These results were corroborated by the *in vitro* data that showed increased α-SMA expression of cells attached to the PDGF-BB LbL rods in time, but to a lesser extent than TGF-β LbL rods, and only increased GAGs and elastin synthesis in the first days. Moreover, tissue capsules formed onto PDGF-BB LbL rods displayed parts with many leukocytes. In contrast to our previous study^8^ where a matrix- and cell-rich noninflammatory tissue capsule was obtained in a 3 week implant period, PDGF-BB containing LbL rods might have evoked a prolonged inflammatory response due to the leukotactic character of PDGF-BB.^58,59^ After 3 weeks, the fibrotic response seemed to be still active, as myofibroblasts, the key cells in fibrotic response,^49^ were Ki67 positive and hence still proliferated.^60^ Exogenous delivery of growth factors involved in the foreign body response can manipulate inflammatory response and shift the wound-healing trajectory.^61^ This could be useful to hypothesize that secretion of necessary proteins such as collagen and elastin might require more than 3 weeks to be optimally produced. Moreover, the presence of leukocytes might be beneficial in secreting high-mobility group box 1, which has been revealed to control the nutrients and oxygen supply of regenerating tissues, and vascularization would be jeopardized in its absence.^62^

### IGF-1 Releasing Implants

IGF-1 is a mitogen for smooth muscle cells and fibroblasts^63^ and is reported to upregulate tropoelastin mRNA.^64^ Therefore, little α-SMA expression was seen *in vitro* as expected. *In vivo*, only the outer layer contained some myofibroblasts; compared to control nonreleasing rods, the tissue capsule was somewhat thicker. The IGF-1 LbL release did not upregulate elastin synthesis. However, most studies that reported elasto-inductive effects of IGF-1 used embryonic or neonatal cells^64−66^ with a higher elastogenic potential than adult cells.^67,68^ This may explain the lack of elastogenesis.

### Dual-Releasing Implants

The combined release of TGF-β1/PDGF-BB showed a synergistic effect of both single-release LbL rods. Tissue capsules were composed of a mixture of fibroblasts and myofibroblasts, which was supported by *in vitro* α-SMA staining. In line with this, matrix synthesis *in vitro* was decreased compared to TGF-β1 single release. In contrast, TGF-β1/IGF-1 LbL rods generated similar tissue capsules as single IGF-1 release LbL rods, even though the amount of released TGF-β1 was comparable in single and dual releases. However, the total amount of IGF-1 released in dual release was higher than the total amount of IGF-1 from the single release. In addition, these effects may be attributed to the mitogenic activity of IGF-1.^63^

### Elastogenesis

Elastin is a critical structural and regulatory matrix protein in blood vessels and is a missing link in vascular tissue engineering.^69^ Although elastin was scarcely present *in vivo*, *in vitro* studies showed an increase in cell elastin secretion for LbL discs with the exception of PDGF-BB LbL alone, which could be expected as both IGF-1^70^ and TGF-β1^10,45^ have been shown to enhance elastin secretion. Remarkably, the highest total elastin secretion was seen in the TGF-β1/PDGF-BB combination, which is likely caused by the higher cell attachment in TGF-β1/PDGF-BB LbL discs, and hence a higher total secretion of elastin. This shows the capability of LbL discs to induce elastin production. The lack of effect on elastin synthesis *in vivo* might be due to the shift of the wound response trajectory as mentioned before, hence taking more time before sufficient elastin is generated. Furthermore, the formation of functional elastic fibers is a very complex process, which involves the production of other proteins such as fibulin and lysyl oxidase-mediated cross-linking.^71^ Longer time points in the *in vivo* studies might reveal if indeed this is the case. If cells are actively proliferating, as observed in our study, they are less prone to synthesizing new proteins. This hinders elastin protein synthesis.^41^ In addition, proteases such as cathepsins, MMPs, and their endogenous inhibitors as well as tissue inhibitors of matrix metalloproteinases determine the net balance between matrix synthesis and degradation. Blocking the negative elastin regulator microRNA-29a might be a promising approach to combine with positive regulators such as IGF-1 and TGF-β1,^72^ to further enhance elastogenesis. In previous studies,^73^ we developed a TEBV with burst pressure well above the required values but had relatively low compliance due to the lack of sufficient elastin. Using optimized LbL implants, such as the combination of TGF-β1/PDGF-BB, which could promote elastogenesis, and TGF-β1 for collagen and elastin synthesis per cell, rather than chloroform-/oxygen-treated (control) implants alone, might improve compliance as well.

Our study shows how the use of the LbL technique could steer the composition of the fibrocellular tissue capsule formed from FBR for the *in situ* engineering of TEBVs. From the studied conditions, we could not make a univocal choice on the most optimal GF delivery. Elastin and collagen syntheses were enhanced with TGF-β1 delivery, with collagen also enhanced by IGF-1 delivery, whereas PDGF-BB burst delivery and TGF-β1/IGF-1 dual delivery supported increased vimentin secretion. In future studies, we should also investigate the dual delivery of PDGF-BB/IGF-1, which is known to have a synergistic effect on ECM synthesis. It is also to be noted that we do not know if the observed synthesized elastin is cross-linked or not. For the functionality of elastic fibers, cross-linking is pivotal. This process is very complex and, for instance, requires a good balance between tropoelastin and lysyl oxidase. In future studies, it would be interesting to further examine the state of elastin, which could be achieved, for example, by magnetic resonance imaging *in vivo*.

## Conclusions

In summary, we have evaluated LbL-containing growth factor implants in an *in vivo* bioreactor approach using a rat model and examined tissue composition and cell population in both *in vitro* and *in vivo* studies. The formed tissue capsules have circumferentially orientated fibrillar collagen and GAGs, with no systematic uptake of the loaded growth factors. Our optimized LbL method showed high capability in maintaining an efficient release of growth factors *in vitro* by enhancing myofibroblast differentiation. Myofibroblast-rich tissue capsules of TGF-β1 LbL rods provided the best stimulation for a potential tissue capsule that differentiated and developed into a well-designed TEBV upon vasculature grafting and exposure to high blood flow. TGF-β1/PDGF-BB discs supported the highest cellular adhesion and displayed the highest amount of total elastin. TGF-β1 discs provided the highest collagen and elastin synthesis per cell, compared to chloroform-treated (control) rods. Hence, these selected LBL implants might demonstrate improved compliance in a large animal model implantation. By combining the controlled growth factor release through LbL polyelectrolyte coatings and the surface-enhanced *in vivo* bioreactor strategy, improved functionality can be provided to TEBVs.

## Supporting Information Available

Growth factor concentration before implantation and after explantation; number of cells (per 1000 μm^2^) presenting the transmembrane glycoprotein CD68 and the C-type lectin protein domain CD206; cell attachment and metabolic activity on days 1, 4, and 7; *in vitro* SEM image showing different cell distributions and morphologies on day 1; total collagen and elastin secretion; *in vitro* glycosaminoglycan measurement normalized by cells attached on days 1, 4, and 7; and overview of implanted rods in the subcutaneous rat model (PDF)
