# Supplementary material for: Long-Term Controlled Growth Factor Release Using Layer-by-Layer Assembly for the Development of In Vivo Tissue-Engineered Blood Vessels
Source: ACS Appl Mater Interfaces. 2022 Jun 13;14(25):28591–603. doi: 10.1021/acsami.2c05988 (PMC9247980; doi:10.1021/acsami.2c05988)
Supplement: Supplementary file 1 — am2c05988_si_001.pdf [file am2c05988_si_001.pdf]

Supporting Information

Long-term controlled growth factor release using layer-by-layer assembly for the development of *in vivo* tissue engineered blood vessels

Febriyani F. R. Damanik[1,3,#], Carolien T. Rothuizen[2,#], Reshma Lalai[2], Sandhia Khoenkhoen[3], Clemens van Blitterswijk[1,4], Joris I. Rotmans[2] & Lorenzo Moroni[1,4,*]

1. University of Twente, Drienerlolaan 5, Zuidhorst 145, 7522 NB Enschede, the Netherlands
2. Department of Internal Medicine, Leiden University Medical Center, PO-box 9600 2300 RC Leiden, The Netherlands
3. Radboud University, Faculty of Science, Heyendaalseweg 135, 6525 AJ Nijmegen, The Netherlands
4. Maastricht University, MERLN Institute for Technology Inspired Regenerative Medicine, Complex Tissue Regeneration, PO Box 616, 6200 MD Maastricht, The Netherlands

# F.F.R.D and C.T.R. contributed equally to this work
* Corresponding author: Lorenzo Moroni, l.moroni@maastrichtuniversity.nl

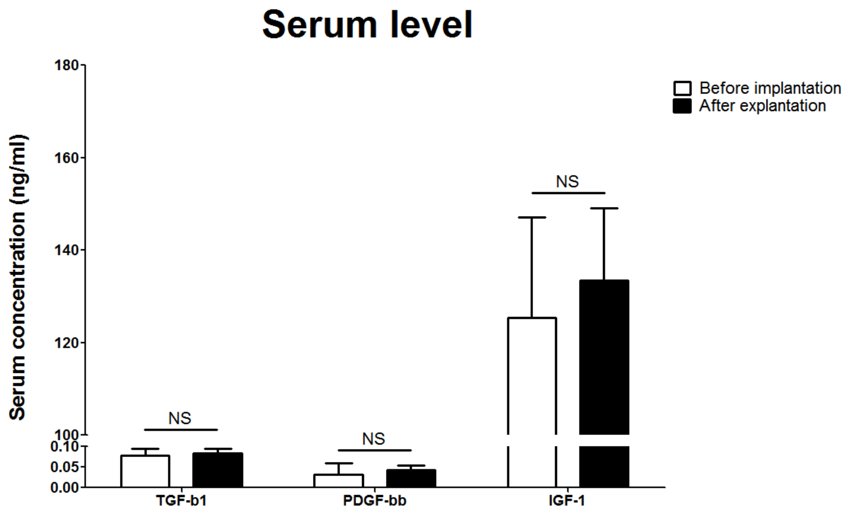

Figure S1. Growth factors concentration before implantation and after explantation.

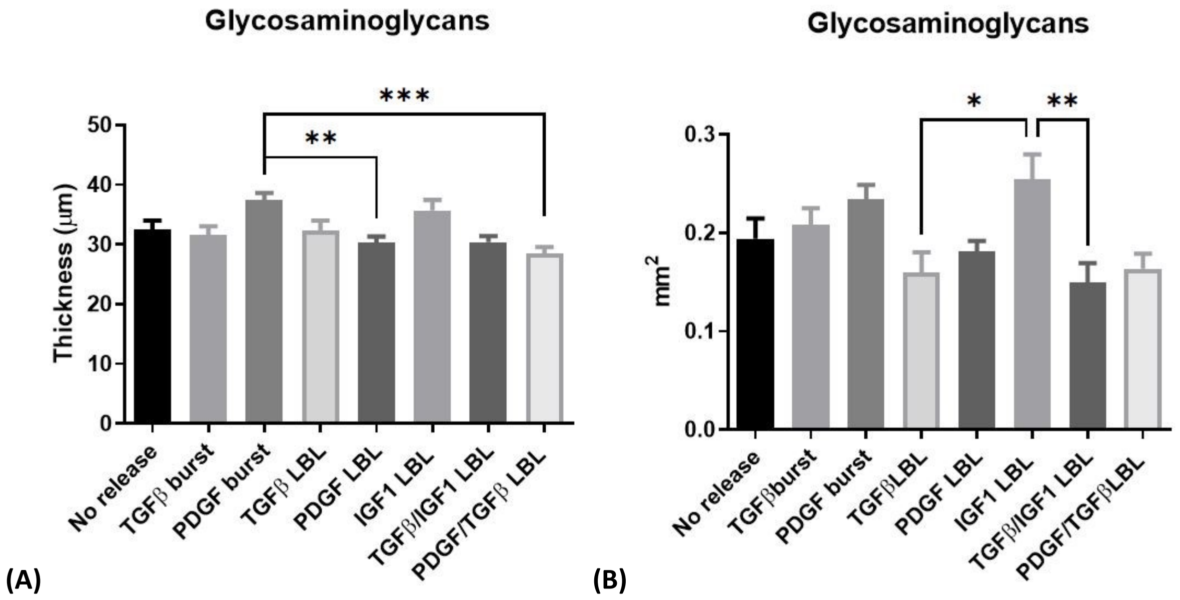

Figure S2. (A) The thickness (in μm) of positively stained ECM with alcian blue for glycosaminoglycans (GAG's). (B) The total area (in mm^2) positively stained with alcian blue for GAG's is depicted. The expression of GAG's was observed in the following conditions: control (no release), burst release (TGF-β1 and PDGF-BB), single layer-by-layer release (TGF-β1, PDGF-BB and IGF1) and dual layer-by-layer release (TGF-β1/IGF1 and PDGF-BB/TGF-β1). Data was analysed using ordinary one-way ANOVA followed by a post-hoc analysis using Tukey's test and * indicates significance of $P \leq 0.05$, $^{**}P \leq 0.01$, $^{***}P \leq 0.001$ and $^{****}P \leq 0.0001$.

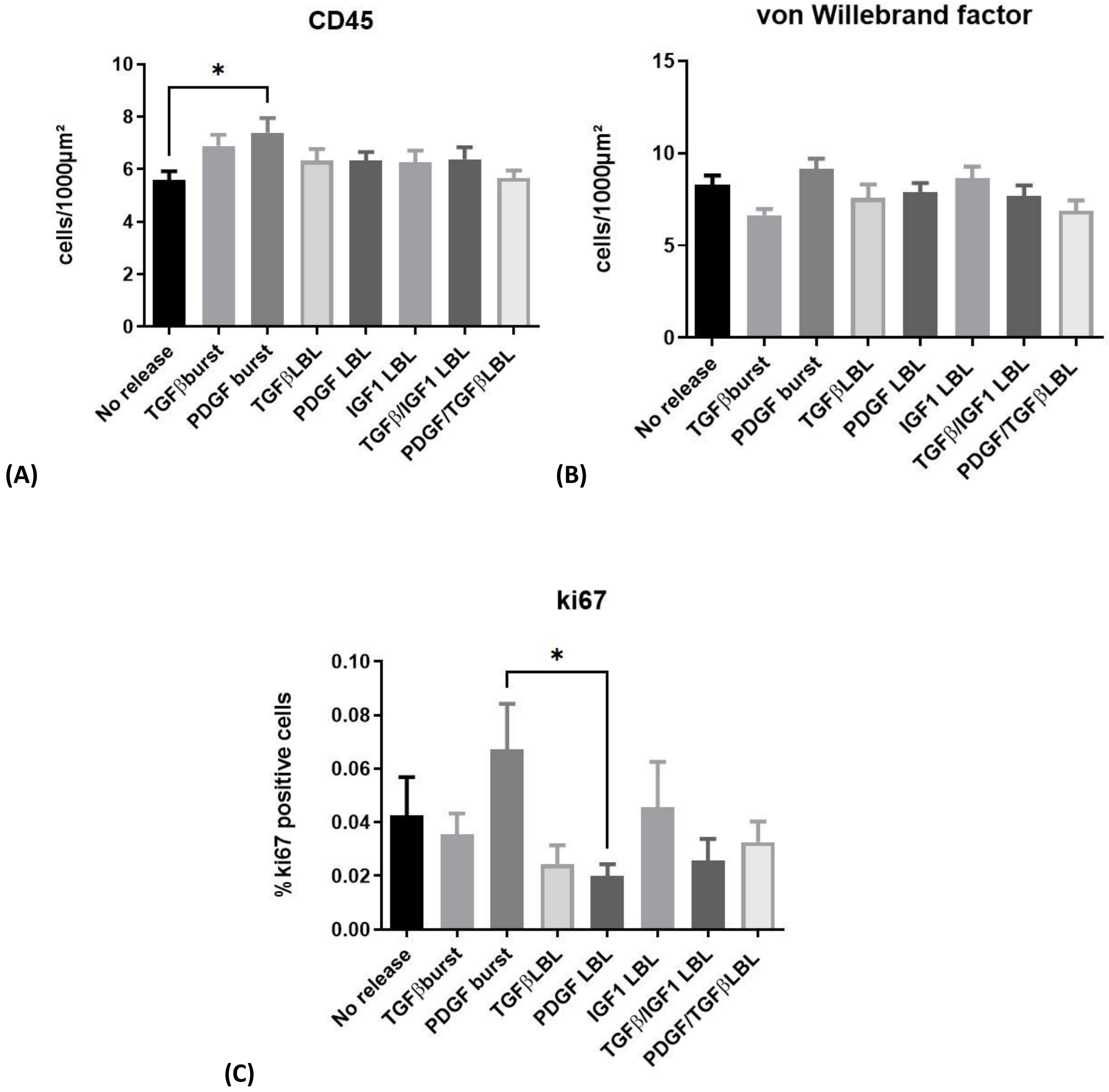

Figure S3. (A) The number of cells (per 1000 μm²) present with the transmembrane glycoprotein CD45 is displayed. (B) The number of cells (per 1000 μm²) positively stained for the von Willebrand Factor is depicted, which presents the vascular endothelial cells. (C) The percentage of proliferating cells is depicted. The expression of leucocytes, endothelial cells and proliferating cells were observed in the following conditions: control (no release), burst release (TGF-β1 and PDGF-BB), single layer-by-layer release (TGF-β1, PDGF-BB and IGF1) and dual layer-by-layer release (TGF-β1/IGF1 and PDGF-BB/TGF-β1). Data was analysed using ordinary one-way ANOVA followed by a post-hoc analysis using Tukey's test and * indicates significance of $P \leq 0.05$.

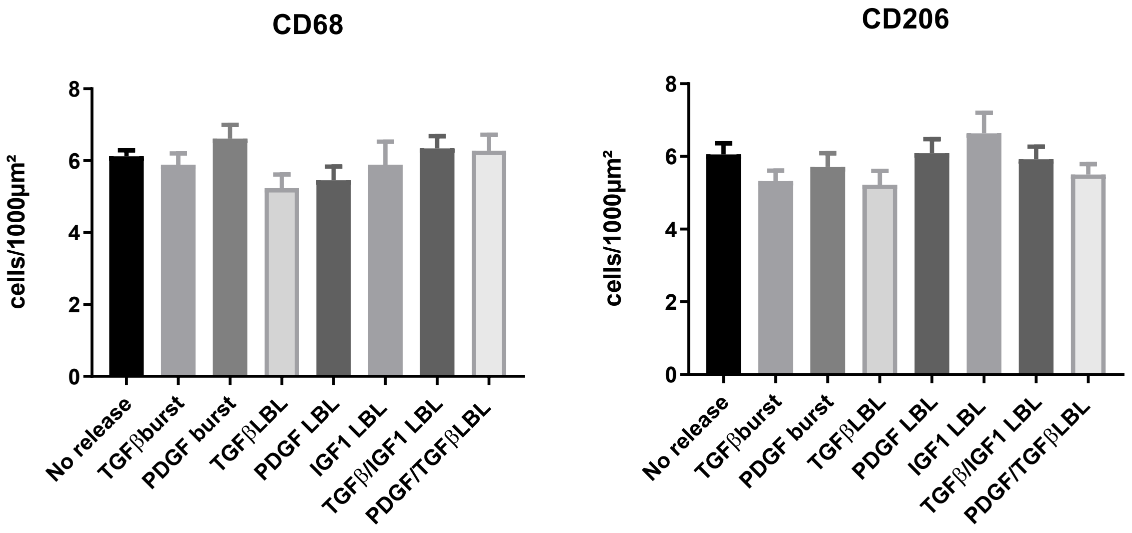

Figure S4. The number of cells (per 1000 µm²) presenting the transmembrane glycoprotein CD68 (left) and the C-type lectin protein domain CD206 (right). The expression of monocytes and macrophages were observed in the following conditions: control (no release), burst release (TGF-β1 and PDGF-BB), single layer-by-layer release (TGF-β1, PDGF-BB and IGF1) and dual layer-by-layer release (TGF-β1/IGF1 and PDGF-BB/TGF-β1). Data was analysed using ordinary one-way ANOVA followed by a post-hoc analysis using Tukey's test. No significances were found.

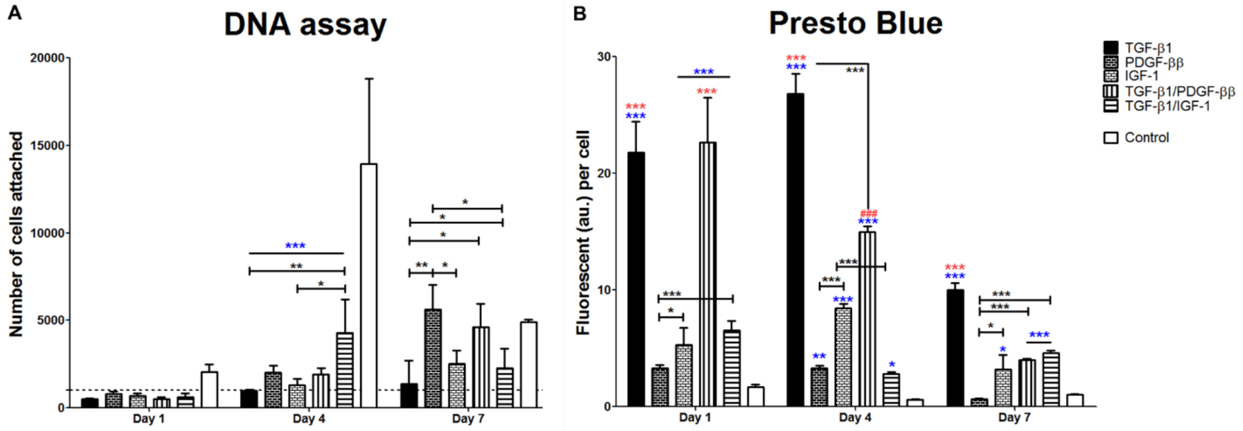

Figure S5. Cell attachment and metabolic activity at day 1, 4 and 7. Different discs are represented by different bar patterns. Data are shown as mean ± s.d. (n = 3 for DNA, and n = 6 for Presto Blue). (A) DNA assay at day 4 shows statistically significant differences when comparing LbL discs with control. At day 7, cells found in PDGF-BB containing discs were higher than other LbL discs. (B) Presto blue assay shows highest metabolic activity in cells attached to TGF-β1 releasing discs and higher metabolic activity in cells found in all LbL discs than in control in all time points with exception to PDGF-BB discs. Blue stars (*$P < 0.05$, ** $P < 0.01$, *** $P < 0.001$) indicate statistical significances in comparison to control, red stars indicate the best parameter from all the treatment types, with red # show second best, while black stars evaluate statistical differences between the different discs.

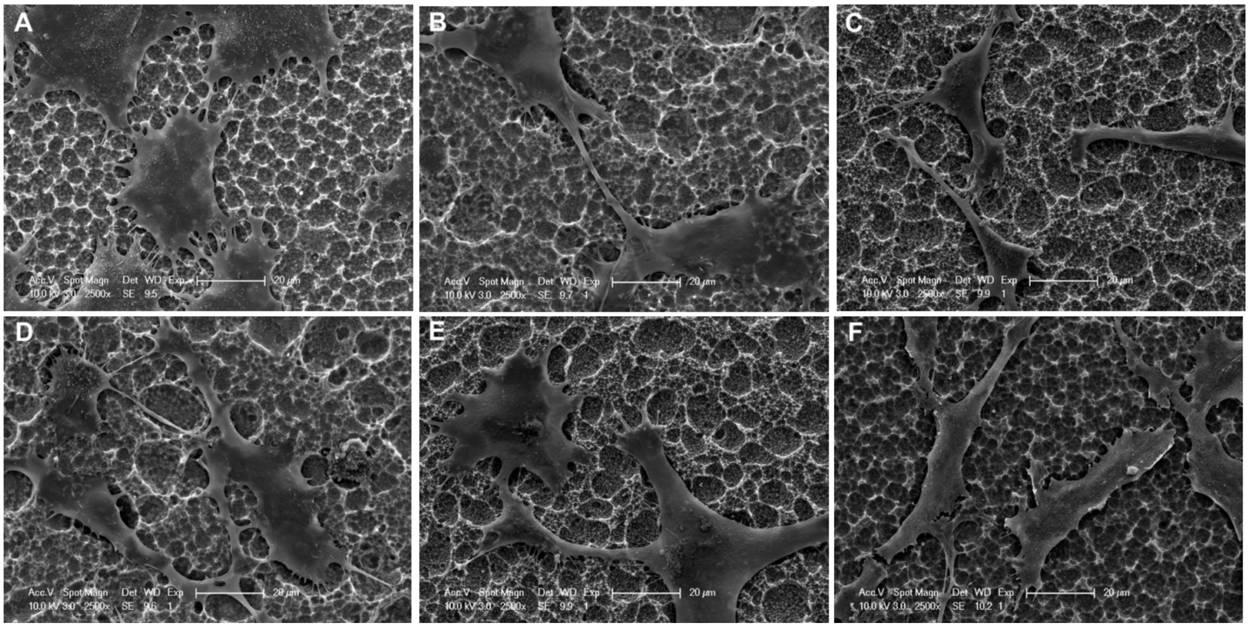

Figure S6. *In vitro* SEM image show different cell distribution and morphology at day 1. (A) Cells on TGF-β1 discs seem to have attached in a square like structure, while cells on (B) PDGF-BB and (C) IGF-1 spread in an elongated manner, similar to (F) and control. (D, E) Cell attached to TGF-β1/PDGF-BB and TGF-β1/IGF-1 discs showed both squared and elongated cell morphology. Scale bar: 20 μm.

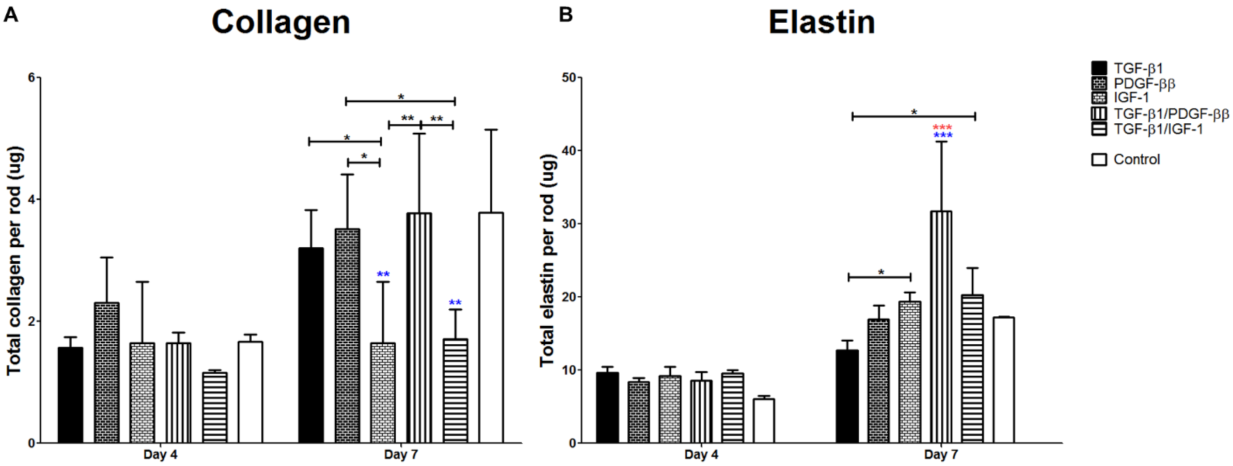

Figure S7. Total collagen and elastin secretion. Different rods are represented by different bar patterns. Data are shown as mean ± s.d. (n = 3). (A) Collagen assay showed significantly lower collagen secretion by cells in IGF-1 discs containing LbL at day 7. Similar total quantity of collagen secreted were seen by cells found in other LbL discs at day 7 and all LbL discs at day 4, compared to control. (B) Highest total elastin secretion was seen in cells in TGF-β1/ PDGF-BB discs at day 7, being statistically significant compared to control. Blue stars (*P < 0.05, ** P < 0.01, *** P < 0.001) indicate statistical significances in comparison to control, red stars indicate the best parameter from all the treatment types, with red # show second best, while black stars evaluate statistical differences between the different discs**.**

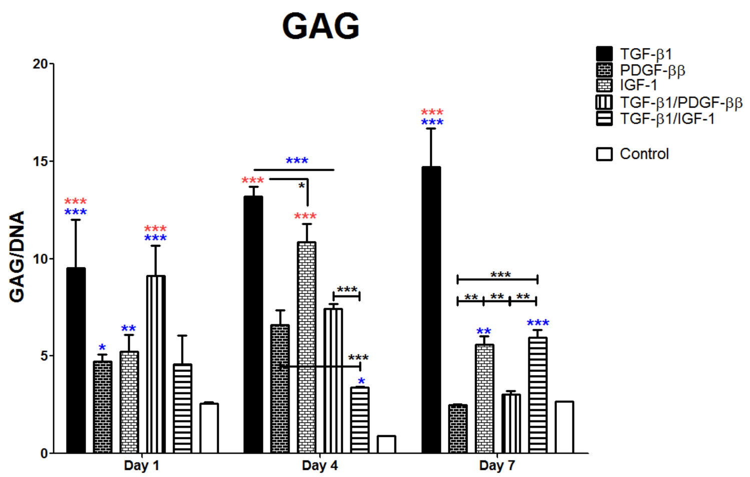

Figure S8. ***In vitro*** **glycosaminoglycan measurement normalized by cells attached at day 1, 4 and 7.** Different discs are represented by different bar patterns. Data are shown as mean ± s.d. (n = 3). GAG secretion per number of cells was highest in TGF-β1 discs in all time points. LbL discs significantly secreted more GAG than control at day 1 and 4, while being significantly higher only in TGF-β1 and IGF-1 containing LbL discs at day 7. Blue stars (*P < 0.05, ** P < 0.01, *** P < 0.001) indicate statistical significances in comparison to control, red stars indicate the best parameter from all the treatment types, with red # show second best, while black stars evaluate statistical differences between the different discs**.**

Table S1. Overview of implanted rods in the subcutaneous rat model.

Name rod	Composition	Modification	Coating	Concentration
Pa300 control	300PEOT$_{55}$PBT$_{45}$	oxygen 100W + chloroform etching	none	-
Pa300 TGF-β1 burst	300PEOT$_{55}$PBT$_{45}$	oxygen 100W + chloroform etching	TGF-β1, dip-coating	10ng/mL
Pa300 TGF-β1 LbL	300PEOT$_{55}$PBT$_{45}$	oxygen 100W + chloroform etching	TGF-β1, LbL	10ng/mL
Pa300 PDGF-BB burst	300PEOT$_{55}$PBT$_{45}$	oxygen 100W + chloroform etching	PDGF-BB, dip-coating	40ng/mL
Pa300 PDGF-BB LbL	300PEOT$_{55}$PBT$_{45}$	oxygen 100W + chloroform etching	PDGF-BB, LbL	40ng/mL
Pa300 TGF/PDGF	300PEOT$_{55}$PBT$_{45}$	oxygen 100W + chloroform etching	TGF- β1 LbL + PDGF-BB LbL	10ng/mL + 40ng/mL
Pa300 IGF-1	300PEOT$_{55}$PBT$_{45}$	oxygen 100W + chloroform etching	IGF-1 LbL	500ng/mL
Pa300 TGF/IGF	300PEOT$_{55}$PBT$_{45}$	oxygen 100W + chloroform etching	TGF- β1 LbL + IGF-1 LbL	10ng/mL + 500ng/mL
